# ACE2: Evidence of role as entry receptor for SARS-CoV-2 and implications in comorbidities

**DOI:** 10.7554/eLife.61390

**Published:** 2020-11-09

**Authors:** Natalia Zamorano Cuervo, Nathalie Grandvaux

**Affiliations:** 1 CRCHUM - Centre Hospitalier de l’Université de MontréalQuébecCanada; 2 Department of Biochemistry and Molecular Medicine, Faculty of Medicine, Université de MontréalQuébecCanada; Radboud University Medical CenterNetherlands; Radboud University Medical CentreNetherlands

**Keywords:** ACE2, SARS-CoV-2, COVID-19, comorbidities, receptor, virus

## Abstract

Pandemic severe acute respiratory syndrome coronavirus 2 (SARS-CoV-2) causes coronavirus 19 disease (COVID-19) which presents a large spectrum of manifestations with fatal outcomes in vulnerable people over 70-years-old and with hypertension, diabetes, obesity, cardiovascular disease, COPD, and smoking status. Knowledge of the entry receptor is key to understand SARS-CoV-2 tropism, transmission and pathogenesis. Early evidence pointed to angiotensin-converting enzyme 2 (ACE2) as SARS-CoV-2 entry receptor. Here, we provide a critical summary of the current knowledge highlighting the limitations and remaining gaps that need to be addressed to fully characterize ACE2 function in SARS-CoV-2 infection and associated pathogenesis. We also discuss ACE2 expression and potential role in the context of comorbidities associated with poor COVID-19 outcomes. Finally, we discuss the potential co-receptors/attachment factors such as neuropilins, heparan sulfate and sialic acids and the putative alternative receptors, such as CD147 and GRP78.

## Introduction

Severe acute respiratory syndrome (SARS)-Coronavirus 2 (SARS-CoV-2) has recently been identified as the causative agent of a new severe respiratory disorder, known as coronavirus 19 disease (COVID-19), which was first detected in Wuhan, China, in December 2019 (Zhu et al., 2020). SARS-CoV-2 belongs to the Coronaviridae family, which includes evolutionary related enveloped (+) strand RNA viruses of vertebrates, such as seasonal common coronaviruses, SARS-CoV and Middle East Respiratory Syndrome (MERS)-CoV, which are responsible for severe clinical syndromes (Lu et al., 2020).

SARS-CoV-2 is highly transmissible (Sanche et al., 2020; Zheng, 2020). By the end of September 2020, SARS-CoV-2 had already infected more than 34 million people worldwide (Worldometers.info, 2020). Transmission occurs primarily through inhalation of droplets emitted from an infected person or through contact with contaminated surfaces on which viruses can remain infectious for several days (van Doremalen et al., 2020). The difficulty in controlling the spread of SARS-CoV-2 is in part due to the unusual shedding of the virus by asymptomatic individuals (Gandhi et al., 2020). While most infected people only experience mild to moderate respiratory symptoms after an incubation period of up to 14 days, others face severe symptoms ultimately leading to acute respiratory distress syndrome (ARDS) associated with a cytokine storm syndrome (CSS) (Wadman and Kaiser, 2020). The pulmonary pathobiology of COVID-19 differs from other severe respiratory infections with typical ground glass opacities and vascular endotheliitis and angiogenesis (Ackermann et al., 2020). Although COVID-19 is a disease initiated in the lungs, it is clear that the symptoms affect other organs and that the most severe cases present multisystem involvement (Wadman and Kaiser, 2020; Varga et al., 2020). Understanding of these disorders outside the lungs is still limited, but probably essential to suggest appropriate treatment strategies. Over 1, 000,000 individuals have died from COVID-19 at the end of September 2020 (Worldometers.info, 2020).

SARS-CoV-2 is closely related to SARS-CoV, which was responsible for a global outbreak in 2003. The high similarities seen in the spike (S) glycoproteins exposed on the surface of virions quickly suggested that SARS-CoV-2 could use the membrane protein angiotensin two converting enzyme (ACE2) as an entry receptor similarly to SARS-CoV (Wan et al., 2020; Li, 2013; Li et al., 2006; Hoffmann et al., 2020; Zhou et al., 2020).

Research on SARS-CoV-2 and COVID-19 is advancing at unprecedented speed. Much data has already been accumulated, some still in the pre-printing stage, that supports ongoing research aimed at understanding pathogenesis and provides information on potential therapeutic strategies. Here, we offer a critical summary of the current knowledge highlighting the remaining gaps that need to be filled to fully characterize the function of ACE2 in the infection by SARS-CoV-2 and the associated pathogenesis. Current evidence supports the low expression of ACE2 in the human respiratory system, which raises questions about the exact role of ACE2 in SARS-CoV-2 infection and has given rise to the hypothesis that co-receptors/attachment factors, such as neuropilins, heparan sulfate, and sialic acids or putative alternative receptors, such as CD147 and GRP78, could be involved in the entry of SARS-CoV-2 and contribute to tropism.

### Evidence supporting ACE2 as an entry receptor for SARS-CoV-2

#### 1.1 In-silico and in-vitro studies supporting interaction between ACE2 and SARS-CoV-2 spike protein

**Figure 1.**
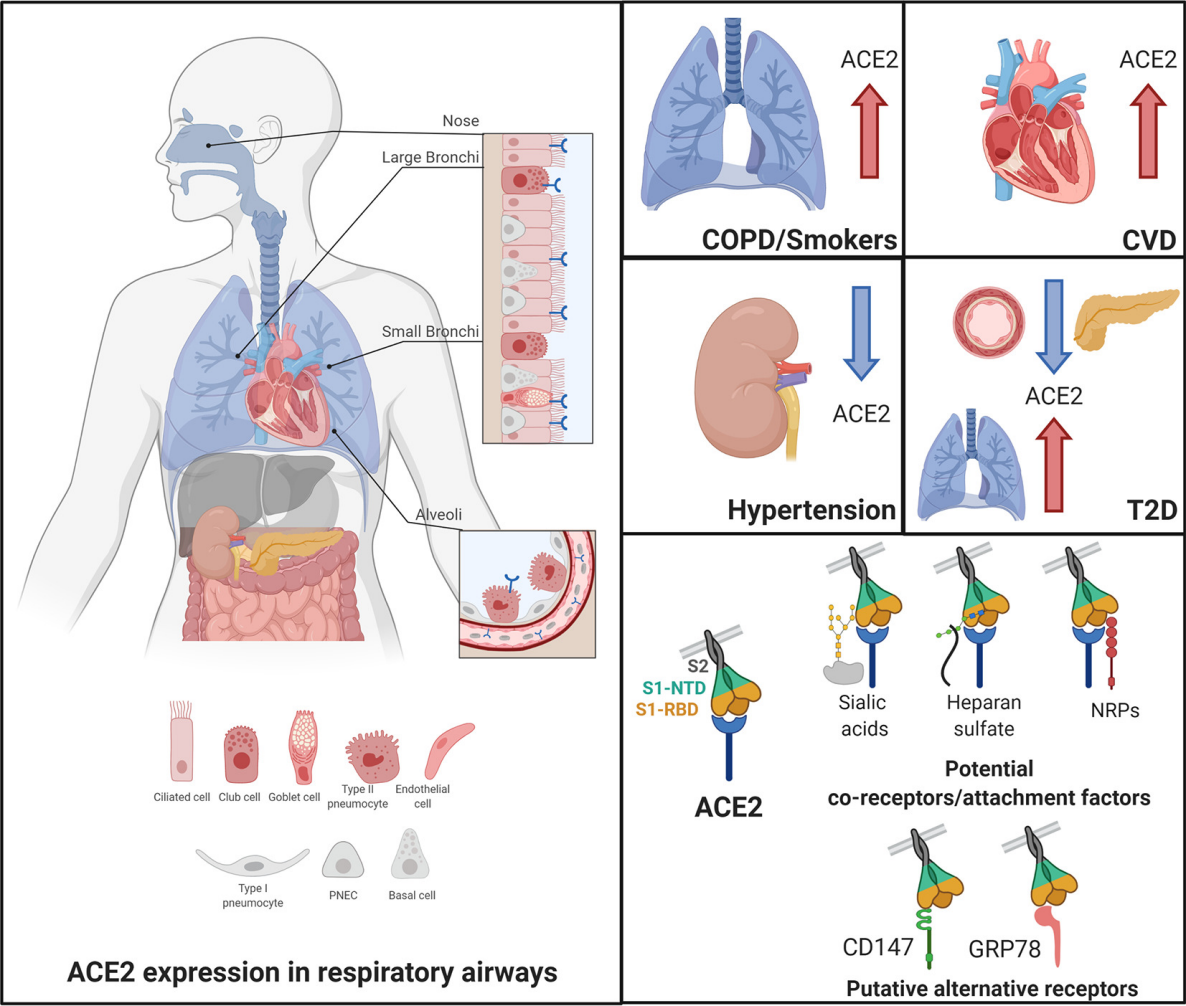
Angiotensin-converting enzyme 2 (ACE2), the proposed receptor of SARS-CoV-2, is expressed in the respiratory airways at low levels (blue) compared to the intestine, kidney, heart, and pancreas. Low levels are also observed in the liver. In nasal and bronchial tissues, ACE2 is mainly expressed by ciliated, club, and goblet cells. It is also found in type-2 pneumocytes of alveoli and in endothelial cells of pulmonary capillaries. In comorbidities associated with a severity and poor prognosis of COVID-19, ACE2 levels are increased in the lungs of COPD and smokers and in the heart of patients with cardiovascular diseases (CVD). In contrast, patients with hypertension exhibit decreased levels of ACE2 in the kidney. In T2D patients, ACE2 is decreased in the pancreas and the vascular system but increased in the lungs. Current evidence supports a possible role of co-receptors or attachment factors, such as neuropilins, heparan sulfate, and sialic acids. The low detection of ACE2 in respiratory tissues also led to the speculation of a role of alternative receptors, such as CD147 and GRP78.

The surface glycoprotein S of coronaviruses mediating the attachment and entry into target cells is composed of 2 subunits, S1 and S2. The S1 subunit contains a N-terminal domain (NTD) and a receptor-binding domain (RBD) encompassing the receptor-binding motif (RBM) (Figure 1). The S2 contains a fusion peptide (FP), heptad repeat 1 (HR1) and 2 (HR2) domains, and a transmembrane (TM) and a cytoplasmic (CP) domain (Xia et al., 2020a; Li et al., 2005; Ou et al., 2020). After S1 binding to a membrane receptor, the FP is inserted into the cell membrane to promote fusion with the viral membrane, a process that depends on proteolytic cleavages at the S1/S2 site to separate S1 and S2 and at the S2 site to generate a mature FP (Hoffmann et al., 2018).

After SARS-CoV outbreak in 2003, ACE2 was identified as the receptor for entry into lung epithelial cells (Li et al., 2003). Homologies between the RBD/RBM of SARS-CoV-2 and SARS-CoV led to the hypothesis that ACE2 could also serve as a receptor for SARS-CoV-2. RBDs share 75% similarities (Wan et al., 2020). Amino acid-based structure-function predictive framework analyses revealed similarities specifically in the hot spot regions responsible for the stability of SARS-CoV binding to ACE2 that may play a role in the zoonotic and human-to-human transmissions (Li, 2013; Li et al., 2006). However, 6 of the 14 amino acids of these hot spots differ in SARS-CoV-2 (L455, F486, Q493, S494, N501, and Y505). A plausible hypothesis is that these residues are the result of natural selection and may contribute to a greater affinity for human ACE2 (Wang et al., 2020c; Andersen et al., 2020).

Subsequent in-vitro studies, notably by resolution of cryo-EM structures, confirmed the structural similarities of the interaction between the S proteins of SARS-CoV and SARS-CoV-2 and ACE2, but also delineated further the divergences (Wan et al., 2020; Li, 2013; Li et al., 2006; Wrapp et al., 2020). The in-depth analysis of the interaction between the peptidase (PD) domain of ACE2 (19–615 aa) and the RBD of SARS-CoV (306–527 aa) highlighted the role of the amino acids involved in polar (A475, N487, E484, Y453), ionic (K417) and hydrophobic (Y489, F486) interactions and hydrogen bonds (G446, Y449, G496, Q498, T500, G502). On the other hand, these interactions are not engaged with the RBD of SARS-CoV-2 (319–541 aa) as predicted in part by the in-silico modeling (Wan et al., 2020; Wang et al., 2020a; Ortega et al., 2020). Additionally, the X-ray crystallographic structure of the PD domain (19–615 aa) in complex with the RBDs (319–541 aa) suggests the involvement of K417, G446, A475, and Q493 in the interaction with SARS-CoV-2, but not with SARS-CoV (Lan et al., 2020). A limitation of these studies is the use of ACE2 fragments lacking the C-terminal collectrin (CLD) and TM-like domains, which probably affects the interaction with the S protein. The cryo-EM analysis of the complete recombinant ACE2 suggests that the dimer coexists between an open and a closed state, while in the presence of the RBD of SARS-CoV-2 (319–541 aa) only the closed conformation is observed (Yan et al., 2020). This study also highlighted the involvement of K417 and G446 in the ACE2/SARS-CoV-2 interaction. As expected, one RBD domain interacted with the PD domain of one ACE2 monomer with an interface similar to the one found with SARS-CoV S (Wang et al., 2020a). The structure of the full-length SARS-CoV-2 S determined by Walls et al. also revealed open and closed conformational states, which were not observed using the 319–541 aa fragment (Yan et al., 2020; Walls et al., 2020). This structure also demonstrated the role of F486 and N501, which were predicted in silico, and of K417 in the interaction with ACE2 (Wan et al., 2020).

Several reports using surface plasmon resonance (SPR) to study the interaction between recombinant ACE2 and S proteins have determined a higher Kd for SARS-CoV S than for SARS-CoV-2 S (Table 1). Although these studies point to higher affinity of ACE2 for SARS-CoV-2 S than for SARS-CoV S, they are in contrast with results obtained using a biolayer interferometry binding (BLI) approach (Table 1). At this point, one cannot exclude that these discrepancies result from the analysis of distinct domains. Additionally, currently available measures rely only on protein fragments lacking the TM domains that likely alter the dynamics of the three-dimensional protein structure, prompting a careful interpretation of the available data.

**Table 1.** Measure of the dissociation constant (Kd) of ACE2 bound to immobilized SARS-CoV or SARS-CoV-2 S proteins by surface plasmon resonance (SPR) or biolayer interferometry binding (BLI) approaches.

Reference	ACE2 protein PD domain	SARS-CoV S	SARS-CoV2 S	Method	Measured kd
Wrapp et al., 2020	1–615 aa	306–577 aa		SPR	325.8 nM
			1–1208 aa		14.7 nM
Wang et al., 2020a	19–615 aa	306–527 aa		SPR	408.7 nM
			319–541 aa		133.3 nM
Lan et al., 2020	19–615 aa	306–527 aa		SPR	31.6 nM
			319–541 aa		4.7 nM
Walls et al., 2020	1–614 aa	306–575 aa		BLI	1.2 nM
			328–533 aa		5 nM
Wrapp et al., 2020	1–615 aa	306–577 aa		BLI	13.7 nM
			319–591 aa		34.6 nM

Overall, in-silico predictions and structural analyses support an interaction between ACE2 and SARS-CoV-2 S with additional contact points compared to the interaction with SARS-CoV S, which are associated with a higher affinity that may explain the easy human-to-human spread (Wrapp et al., 2020). Future studies should aim to further characterize the interaction between full-length ACE2 and SARS-CoV-2 proteins to provide the most precise ground for the development of therapies aimed at disrupting this interaction.

#### 1.2 In cellulo evidence of ACE2 acting as SARS-CoV-2 entry receptor

The extraordinary speed of research following the identification of SARS-CoV-2, particularly the development of pseudotyped viruses (Hoffmann et al., 2020; Ou et al., 2020; Walls et al., 2020; Tai et al., 2020; Lei et al., 2020a), the isolation of the virus from patient samples and the establishment of an in-vitro culture as early as January 2020 (Zhou et al., 2020), quickly made it possible to tackle the question of the identity of the entry receptor in cellular models (Hoffmann et al., 2020). Pseudotyped vesicular stomatitis virus expressing SARS-CoV-2 S (VSV-SARS-S2) efficiently infects only a limited number of cell lines, with Calu-3 human lung adenocarcinoma epithelial cell line, CaCo-2 human colorectal adenocarcinoma colon epithelial cell line and Vero African grey monkey kidney epithelial cell line being the most permissive (Hoffmann et al., 2020; Ou et al., 2020). While permissive cell lines all express ACE2, as previously demonstrated by indirect immunofluorescence (IF) or by immunoblotting (IB, antibody AF933) (Ren et al., 2006; Tseng et al., 2005; Liao et al., 2013; Lu et al., 2008), the role of ACE2 in virus entry was demonstrated by the observation that its ectopic expression is sufficient to make the BHK-21 fibroblast and HEK293 cell lines permissive to VSV-SARS-S2 (Hoffmann et al., 2020; Ou et al., 2020). Importantly, the preincubation of VSV-SARS-S2 with the soluble form of ACE2 prevents the infection likely by blocking the site of S protein interaction. The use of VSV pseudovirions expressing the SARS-CoV S protein (VSV-SARS-S) modified to contain the RBD from other coronaviruses, including MERS-CoV and SARS-CoV-2, allowed to underscore the importance of the RBD in the interaction with ACE2 in cellular models (Letko et al., 2020). Murine leukemia viruses (MLV)-based pseudoviruses expressing SARS-CoV and SARS-CoV-2 S were also found to infect Vero E6 cells (Walls et al., 2020), which express endogenous ACE2 (Sheahan et al., 2008), and ACE2-transfected BHK-21 cells (Walls et al., 2020).

Although pseudotyped viruses are a great tool to document the need for ACE2 for the entry steps mediated by the S protein, they do not make it possible to assess the contribution of other virions characteristics, such as envelope or membrane proteins, on the cell tropism (Joglekar and Sandoval, 2017). Access to SARS-CoV-2 patient isolates allowed to further evaluate the need of ACE2 for virus entry. Indeed, infection of HeLa, BHK-21 or A549 cells with isolates requires transfection of ACE2 (Hoffmann et al., 2020; Blanco-Melo et al., 2020; Zhou et al., 2020). While direct protein interaction in cellular model is difficult to assess, cell surface colocalization of ectopically expressed GFP-tagged ACE2 and SARS-CoV-2 S protein fragments containing the RBD was observed (Wang et al., 2020a). GFP-ACE2 expressed at the cell surface was also shown to bind soluble SARS-CoV-2 RBD; an interaction that was inhibited by incubation with a soluble form of ACE2 (Tai et al., 2020). Collectively, evidence obtained using cell lines further supports in-silico data that ACE2 binds to SARS-CoV-2 S and points to a model in which ACE2 is necessary for virus entry. More physiologically relevant studies are awaited to strengthen our understanding of the exact role of ACE2 in vivo and help determine if co-receptors or attachment factors act in concert with ACE2 and if alternative receptors may play a role in SARS-CoV-2 entry.

### ACE2 expression in the respiratory apparatus

#### 2.1 Basal expression of ACE2 in the human respiratory system

ACE2 was first identified in 2000 as a gene encoding a single isoform of a new angiotensin enzyme (Donoghue et al., 2000). Recently, due to the great interest generated around ACE2, bioinformatics and RNA-seq analyses have revisited the human ACE2 genomic region and identified the existence of an alternative splice site (Rehman and Tabish, 2020; Blume et al., 2020) that results in the transcription of a previously unknown isoform of ACE2 (deltaACE2 or dACE2). The dACE2 isoform lacks the first N-terminal 356 aa region compared to full-length ACE2 (flACE2) (Blume et al., 2020; Onabajo et al., 2020) and therefore is unable to bind to SARS-CoV-2 S (Onabajo et al., 2020). Considering the recent discovery of dACE2, the majority of ACE2 mRNA analyses reported below have not taken into account its existence. However, when possible, we indicated when the design discriminated specific ACE2 isoforms.

High-throughput gene promoter activity and mRNA expression data available in public repositories, that is functional annotation of the mammalian genome (FANTOM 5, flACE2), the Human Protein Atlas (HPA) and the genome‐based tissue expression consortia (GTEx), provide evidence of ACE2 mRNA expression in the respiratory system (Yu et al., 2015; Uhlén et al., 2015; Keen and Moore, 2015). As pointed out in two recent reports, levels of ACE2 mRNA in the respiratory system are low compared to other organs such as the small intestine, kidney or the myocardium (Table 2; Hikmet et al., 2020; Aguiar et al., 2020). An additional transcriptomic dataset of various human tissues confirmed ACE2 mRNA expression throughout the respiratory tract, including fetal and adult lungs, olfactory bulbs and trachea, with the highest expression in the lung (Leung et al., 2020). RNA-seq and microarray datasets from nasal and bronchial epithelial cells obtained through brushings demonstrate ACE2 detection with low levels in the nose, trachea and small and large airways (Leung et al., 2020; Su et al., 2004). Low expression of ACE2 (flACE2) in the nasal respiratory epithelium and at even lower levels in the trachea, bronchioles and alveoli was confirmed by RNA in-situ hybridization (RNA-ISH) of lung and nasal sections (n = 7) (Hou et al., 2020). RT-qPCR of brush samples from the same donors confirmed a gradient of ACE2 mRNA with the strongest expression in the nasal tissue and the lowest in the bronchioles and alveoli (Hou et al., 2020). It is noteworthy that this gradient of ACE2 expression mirrors the profile of permissiveness to SARS-CoV-2. Indeed, infection of primary cells led to higher viral titers in cells from the nasal epithelium and from the large airways than in cells from lower airways or Alveolar type II cells (AT2). Analysis of the specific expression of dACE2 and flACE2 in lung explants and nasal tissue reported similar expression profile at basal levels (Blume et al., 2020; Onabajo et al., 2020). Further analyses are needed to evaluate levels of expression of both isoforms in other respiratory tissues. While altogether these studies provide clues for ACE2 mRNA expression in the respiratory apparatus that is the primary target by SARS-CoV-2, they also shed light on the overall low levels detected at basal levels when performing bulk analyzes of the whole tissues (Figure 1).

**Table 2.** mRNA levels of ACE2 found in the lungs, small intestine, kidney, and heart muscle reported in the Human Protein Atlas (HPA) consortium (Uhlén et al., 2015), the genome‐based tissue expression (GTEx) consortium (Keen and Moore, 2015). Activity levels of the promoter of ACE2 assembled in the Fantom (FANTOM5) consortium (Yu et al., 2015). Protein-transcripts per million (pTPM). Scaled Tags Per Million (sTPM).

	Lung	Small Intestine	Kidney	Heart Muscle
HPA (pTPM)	1.7	31.1	107.2	31.1
GTEx (pTPM)	1.1	5.4	6.8	5.4
FANTOM5 (sTPM)	2.8	21.7	31.5	21.7

The low levels of ACE2 mRNA detected in tissues of the respiratory system likely reflects restricted expression in specific cell subpopulations (Figure 1). This has indeed been documented in several studies using scRNA-seq and snRNA-seq strategies, which make it possible to identify the cell populations expressing ACE2 mRNA. A first report describing the integrated analysis of 107 scRNA-seq and snRNA-seq datasets, including 22 from lungs and airways and 85 from other organs and encompassing 164 donors, revealed ACE2 expression in multi-ciliated epithelial and goblet cells of the proximal airways, and in AT2 cells in the distal lung epithelium (Muus et al., 2020). Expression of ACE2 in AT2 cells (defined as HTII280+ or PRO-SFTPC+ cells) was further confirmed by proximity ligation in-situ hybridization (PLISH) performed on airway and alveoli tissue from healthy donors. A second report describing the re-analysis of five scRNA-seq datasets from the lung, bronchus and nasal tissues, also concluded that ACE2 is enriched in lung AT2 cells and in bronchial and nasal ciliated epithelial and goblet cells (Hikmet et al., 2020). While the two reports led to similar qualitative conclusions, they differed in the quantitative report of the estimated % of ACE2+ AT2 cells, that is 6.2% and less than 1%, respectively. The use of the scRNA-ISH technique that combines scRNA-seq and ISH data, showed 20% of nasal and bronchial cells expressing ACE2 (Okochi et al., 2020). ACE2 mRNA was also detected in a fraction of nasal and bronchial ciliated (FOXJ1+) and secretory (MUC5B+) cells and in AT2 cells (SFTPC+) in the alveoli (Hou et al., 2020).

Several studies attempted to detect ACE2 protein by immunohistochemistry (IHC) in lung histological sections. Results exhibit more discrepancies than mRNA analyses, making it difficult to draw firm conclusions. Significant variations between studies are likely due to poor antibody validation practices, different antigen retrieval methodologies or to low antibody sensitivity. In Table 3, we attempted to summarize the available validation data for the antibodies used in the studies described in this review in accordance to the pillars defined by the Antibodypedia validation initiative (Uhlen et al., 2016). Importantly, all antibodies still await validation by siRNA or CRISPR technologies. Use of HPA000288 and MAB933 antibodies, which allow high detection of ACE2 in kidney and intestine, only showed very low to no detection in lung tissues (Uhlén et al., 2015; Hikmet et al., 2020). Positive staining of ACE2 was only observed in a very small percentage of individuals and corresponded to the surface of nasal ciliated cells, glandular cells of the trachea or AT2 cells consistent with mRNA expression. In a systematic study, six anti-ACE2 antibodies targeting different epitopes, ab15348, HPA000288, ab239924, NBP2-67692, AF933 and MAB933, were tested together with distinct antigen retrieval methods (Lee et al., 2020). Only the ab15348 antibody revealed robust staining in pneumocytes, while as expected the six antibodies showed positive staining in kidney and intestine. Further use of this antibody for indirect IF staining of only one donor revealed ACE2 detection in MUC1 positive type II pneumocytes. Additionally, IHC staining of resected lungs (n = 9) using this same antibody allowed detection of ACE2 in the small airway epithelial layer (Leung et al., 2020). ACE2 was detected in AT2 cells (PRO-SFTPC+) from human explant donors by IHC coupled to IF (IHC-IF) using the AF933, but not ab15348 or MAB933, antibodies, while low counts of ACE2-positive alveolar epithelial cells were detected in post-mortem lung sections (n = 10) using ab108252 (Ackermann et al., 2020; Muus et al., 2020).

**Table 3.** Available validation data for the antibodies used in the studies described in this review in accordance to the pillars defined by the Antibodypedia validation initiative (Uhlen et al., 2016). Provider refers to the information found in the website of the company. IB: Immunoblot. IHC: Immunohistochemistry. IF: Immunofluorescence. 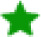 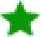
Enhanced validation, 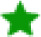
Supportive validation, 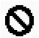
No data available, + Positive detection, +/- Weak detection, - Absence of detection.

	Method	Antibodypedia	Provider	Additional information
Ab15348 (or GTX15348) Immunogen: 788-805aa	IB	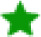	+ Testis, intestines, lung, Calu-3 - Breast	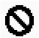
	IHC	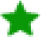	+ Testis, kidney, aorta and lung	+ Intestine, heart, stomach, spleen (Independent antibody validation) (Lee et al., 2020) +, +/-, - Lung. Increased in COPD and smokers (correlation with mRNA) (Leung et al., 2020; Muus et al., 2020; Lee et al., 2020)
	IF	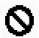	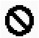	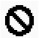
MAB933 Immunogen: 18-74aa	IB	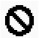	+ Kidney - NSO cells	+ Vero E6 cells - CHO cells (Jia et al., 2005)
	IHC	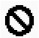	+ Kidney	+ Orthogonal (RNA) and independent antibody validation +/-, - Lung (Hikmet et al., 2020; Aguiar et al., 2020; Muus et al., 2020; Lee et al., 2020)
	IF	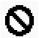	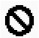	+ ALI-cultured ciliated airway epithelial cells (Martinez-Anton et al., 2013)
AF933 Immunogen: 18-74aa	IB	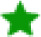	+ Ovary, testis and kidney	+ Airway and distal lung, ALI-cultured airway epithelial cells (correlation with mRNA levels), Calu-3 and Caco-2 cells (Liao et al., 2013; Smith et al., 2020) - A549 (expected), Huh-7 cells (Liao et al., 2013; Smith et al., 2020)
	IHC	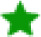	+ Kidney	+ Testis, stomach, intestine (Independent antibody validation) +/-, - Lung (Ren et al., 2006; Muus et al., 2020; Lee et al., 2020)
	IF	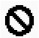	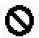	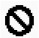
HPA000288 Immunogen: 1-111aa	IB	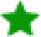	+ Kidney, but several bands	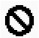
	IHC	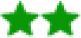	+ Intestine and kidney. - Tonsil	+ Orthogonal (RNA) and independent antibody validation +/-, - Lung (Uhlén et al., 2015; Hikmet et al., 2020; Lee et al., 2020)
	IF	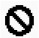	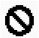	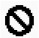
Ab108252 Immunogen: 200-300aa	IB	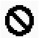	+ Testis, kidney, lung, HepG2, Caco-2 - A549 cells	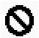
	IHC	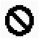	+ Kidney	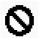
	IF	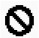	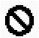	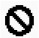
Ab239924 Immunogen: 200-300aa	IB	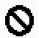	+ Testis, kidney	+ ACE2 transfected A459 - Non transfected A549 cells (Blanco-Melo et al., 2020)
	IHC	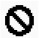	+ Testis, kidney	+ Intestine, heart, stomach, spleen (Independent antibody validation), +/- Lung (Lee et al., 2020)
	IF	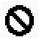	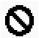	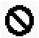
NBP2-67692 Immunogen: 200-300aa	IB	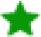	+ Kidney	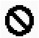
	IHC	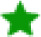	+ Kidney	+ Testis and intestine (Independent antibody validation) - Lung (Lee et al., 2020)
	IF	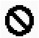	+ HepG2, MCF-7, 293 cells	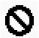
Anti-ACE2^489^ Immunogen: 489-508aa	IB	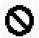	+ACE2 transfected CHO cells - Non transfected CHO cells	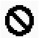
	IHC	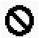	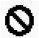	+ Heart (No staining with secondary antibody alone) (Qian et al., 2013)
	IF	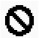	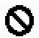	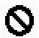
Sc-20998 Immunogen: 631-805aa	IB	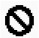	Antibody discontinued	Detection in rat tissue only (Sims et al., 2005)
	IHC	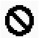	Antibody discontinued	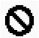
	IF	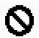	Antibody discontinued	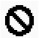
Homemade antibody Immunogen: 206-225aa	IB	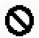	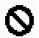	Detection in rat tissue only (Mossel et al., 2008)
	IHC	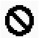	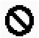	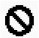
	IF	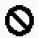	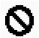	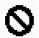

Overall, ACE2 protein is only weakly detected in the respiratory system by two distinct antibodies and a single antibody allows stronger detection in AT2 cells, while all antibodies detect high expression in kidneys and intestine. One cannot fully exclude that ACE2 detection in the lung is impaired by lung-specific post-translational modifications, such as glycosylations, that affects the antigen recognition. Bias from antibodies specificity and sensitivity can be circumvented through proteomic analyses. In this perspective, the low expression of ACE2 in the respiratory system is further supported by the analysis of public mass spectrometry (MS) datasets for protein abundance that does not show detectable ACE2 in the nasal mucosa or the lungs, in contrast to high levels in the kidneys and intestine (Hikmet et al., 2020). Altogether, currently available evidence from mRNA, IHC and MS analyses points to low expression of ACE2 in the respiratory apparatus at basal levels. How these low basal levels of ACE2 expression allow efficient replication and excretion of SARS-CoV-2 is a question that deserves more attention. Moreover, it is still difficult to conclude from the available data that the infection is strictly limited to ACE2 positive cells. These questions remain open and require further investigation.

#### 2.2 ACE2 expression in human respiratory epithelial cells ex vivo: impact of differentiation

Ex vivo culture of cells from the respiratory system is an essential tool for the study of virus infection and pathogenesis. Epithelial cells from distinct sections of the respiratory tract, including the nose, trachea, bronchi and alveoli, are either cultured submerged in culture medium or at air-liquid interface (ALI) for polarization or differentiation into pseudostratified mucociliary epithelium. While submerged culture of human tracheobronchial epithelial cells exhibits low ACE2 mRNA and protein detection by IB(AF933 or MAB933), the expression levels are highly enhanced following differentiation in ALI (Blanco-Melo et al., 2020; Jia et al., 2005; Martinez-Anton et al., 2013; Smith et al., 2020; Qian et al., 2013). More recently, a clear distinction between dACE2 and flACE2 mRNA levels allowed to confirm that both isoforms are increased in differentiated cultures (Blume et al., 2020). Of note, re-submersion of cells, which led to loss of differentiation markers, reversed ACE2 mRNA and protein levels (Jia et al., 2005), supporting a key role of the differentiation process in ACE2 expression. Further study by IF staining of ALI-differentiated human primary alveolar cells (MAB933) showed that ACE2 is localized at the apical surface (Sims et al., 2005). This was recapitulated using confocal horizontal scanning (AF933) of the Calu-3 cell line polarized in ALI (Ren et al., 2006). Importantly, in hTERT-immortalized bronchial cells differentiated into multiciliated cells in ALI, only the flACE2 protein was detected in motile cilia (Blume et al., 2020) suggesting a specific role of the full-length isoform at this location. Overall, these studies show that differentiation significantly increases the expression of ACE2 and, as such, suggests that the use of differentiated cells should be preferred to model ACE2-dependent SARS-CoV-2 infection. Additionally, the heterogeneity of patient-derived respiratory epithelial cells argues for the study of large cohorts to reach clear conclusions. However, this is hardly achievable due to the scarcity of the cells and the high demanding differentiation process. Beside donor to donor variation, IF staining of ALI-cultured AT2 cells also pointed to heterogeneous ACE2 levels in a single culture (Mossel et al., 2008), which correlates with observations made on lung tissue sections showing ACE2 in only a small fraction of the AT2 cells (Muus et al., 2020).

#### 2.3 ACE2 levels during SARS-CoV-2 infection

The correlation between the expression of ACE2 mRNA and that of the classical interferon (IFN) stimulated genes (ISG) that was observed through meta-analysis of scRNA-seq data, as well as the presence of two STAT1 binding sites in the promoter of the human ACE2 gene, supports the hypothesis that ACE2 itself could be an ISG (Ziegler et al., 2020; Hennighausen and Lee, 2020). Several reports have indeed documented the capacity of IFNβ and/or IFNα2 to induce ACE2 mRNA in human basal epithelial cells from nasal scraping, human tracheal cells and human large and small airway cells (Hou et al., 2020; Smith et al., 2020; Ziegler et al., 2020). FlACE2 was also induced in nasal and tracheal cells, but not in small airway cells, in response to IFNγ (Smith et al., 2020). Specific analysis of flACE2 and dACE2 isoforms revealed that in fact IFNα, IFNβ and IFN-λ3 specifically induce dACE2 in human bronchial epithelial cells (HBEC) (Blume et al., 2020; Onabajo et al., 2020). While an increase of dACE2, which does not bind to SARS-CoV-2 S, should not translate into more entry of the virus, the question of how this increase would impact the host antiviral response remains to be determined. The IFN-dependent regulation of ACE2 mRNA expression might explain ACE2 upregulation observed within bystander goblet or squamous cells, but not directly in infected cells, during influenza (IAV) virus infections (Ziegler et al., 2020). Only dACE2 mRNA is upregulated upon IAV and rhinovirus infections, but the role of IFNs in ACE2 upregulation in these contexts remains to be fully addressed (Onabajo et al., 2020).

Converging evidence indicates that ACE2 mRNA levels are also increased during SARS-CoV-2 infection. In a study with a limited cohort, scRNA-seq analysis of nasopharyngeal swabs, bronchial brushes and bronchoalveolar lavages showed up to 3-fold upregulation of ACE2 mRNA in COVID-19 patients (n = 9) compared to healthy individuals (n = 2). ACE2 mRNA induction was observed in secretory and ciliated cells, which also exhibited the most SARS-CoV-2+ cells (Chua et al., 2020). In ALI-differentiated HBEC, SARS-CoV-2-induced ACE2 mRNA is observed in ciliated, basal club and an intermediate between basal cells and club (BC/Club) infected and bystander cells (Ravindra et al., 2020). Surprisingly, this result was not corroborated in the Calu-3 and differentiated BCi-NS1.1 respiratory cell line models, in which neither flACE2 nor dACE2 were induced by SARS-CoV-2 (Blume et al., 2020; Onabajo et al., 2020). This contrasts with the specific induction of dACE2 observed in the non-respiratory Caco-2 cell line (Onabajo et al., 2020). A role of IFNs in the upregulation of ACE2 by SARS-CoV-2 is difficult to conclude based on the current evidence for the status of IFN production and signaling during SARS-CoV-2 infection. In differentiated HBEC, induction of IFNβ and IFNλ mRNA was observed in infected cells, but not in the bystander cells (Ravindra et al., 2020). Increased mRNA levels of IFNβ and IFNλ1 and 3 were also observed in infected Calu-3 cells (Sun et al., 2020; Lei et al., 2020b; Banerjee et al., 2020). This contrasts with the lack of detectable IFNβ or IFNλ1–3 mRNA observed in submerged culture of HBEC upon SARS-CoV-2 infection (Blanco-Melo et al., 2020; Vanderheiden et al., 2020). In an ex vivo human lung explant model, SARS-CoV-2 infection also failed to induce detectable levels of IFNα, β, γ, or λ1–3 at any time point for up to 48 hr, while SARS-CoV induced IFNα, β, γ, or λ1–3 at late time points (Chu et al., 2020). These discrepancies could be due to differences in MOI and time course of the infection, but also from the quality of virus preparation as the content of defective virus particles is known to trigger IFN production in the context of other virus infections (Vignuzzi and López, 2019). Systematic analysis of the impact of these parameters will be necessary to resolve the status of IFN induction by SARS-CoV-2 in respiratory epithelial cells. IFNs plasma levels in COVID-19 patients have also started to be documented. In a first study of critically ill COVID-19 patients, IFN-α2, but neither IFNβ nor IFNλ, was detected in the plasma of 21 out of 26 patients with a peak 10 days after onset of symptoms (Trouillet-Assant et al., 2020). This is in line with a deep analysis of type I IFN mRNA regulation in COVID-19 patients at different stages of the disease. While no IFNβ mRNA and protein were detected in the plasma regardless of the severity of illness (n = 32) compared to healthy donors (n = 13), IFNα mRNA was found in the plasma of all COVID-19 patients (Hadjadj et al., 2020). In two separate reports, plasma levels of IFNα were lower in critically ill patients than in patients with mild to moderate COVID-19 (n = 11 and 10) (Banerjee et al., 2020; Hadjadj et al., 2020). In addition, IFNα levels were maintained in mild to moderate patients for up to 17 days, but decreased in severe and the critically ill patients after 10 and 13 days, respectively (Hadjadj et al., 2020). In addition to evidence supporting weak and shortened levels of IFNα by SARS-CoV-2 infection in critically ill COVID-19 patients, molecular data have started to document the capacity of SARS-CoV-2 to evade the IFN response as previously documented for SARS-CoV (Lei et al., 2020b; Park and Iwasaki, 2020; Xia et al., 2020b). The first evidence results from the overexpression of SARS-CoV-2 proteins in HEK293T cells before infection with Sendai virus or expression of constitutively active RIG-I or MDA5 receptors. Results diverged among the two available reports, but Nsp1, Nsp13, and Orf6 were consistently found to inhibit IFNβ promoter induction and IFN signaling (Lei et al., 2020b; Xia et al., 2020b). The mechanisms underlying these inhibitions are not fully defined, but Nsp13 appears to act upstream of TBK1 phosphorylation, while Orf6 interferes with IRF3 and STAT1 nuclear translocation. A truncation variant of SARS-CoV-2 Orf3b is also thought to be associated with inhibition of IFNβ production (USFQ-COVID19 Consortium et al., 2020). In contrast to the impact of SARS-CoV-2 protein overexpression, SARS-CoV-2 infection of Calu-3 cells, failed to hinder IFNβ induced phosphorylation of STAT1 and STAT2 and downstream IFIT1 production (Banerjee et al., 2020). In summary, the full scope of the interplay between SARS-CoV-2 and the IFN response remains to be carefully characterized before conclusion about the impact on ACE2 induction can be drawn.

Studies documenting ACE2 protein expression upon IFNs stimulation and SARS-CoV-2 infection are really sparse. Immunoblot analysis of ACE2 (AF933) in fully differentiated HBEC derived from asthmatic patients stimulated with IFNγ showed slight increase in only 1 out of 4 donors (Ziegler et al., 2020). In contrast, SARS-CoV-2 infection (MOI of 2) of A549 cells transfected with ACE2 showed decreased levels (ab239924) at 24 hr post-infection (Blanco-Melo et al., 2020). While this is a preliminary observation that needs to be further confirmed, it is in line with the observation that SARS-CoV infection of Vero E6 cells led to downregulation of ACE2 protein levels, which inversely correlated with viral replication (Glowacka et al., 2010). The observation that ACE2 mRNA levels increase or remain stable while the protein levels are decreased during SARS-CoV-2 infection suggests a post-transcriptional regulation mechanism, such as degradation or a halt in the translation. The exact mechanism(s) underlying this dual regulation remains to be investigated to fully comprehend how cells respond to SARS-CoV-2 infection and the potential role of ACE2. These results contrast with the post-mortem examination of lungs from deceased COVID-19 patients (n = 7) compared to age-matched uninfected individuals (n = 10) by IHC (ab108252) which showed a significantly greater number of ACE2-positive alveolar epithelial cells in COVID-19 individuals compared to controls (Ackermann et al., 2020).

Altogether, for the time being, it appears clear that ACE2 mRNA is detected in the respiratory system, but at significantly lower levels than in kidneys and intestine. Detection of ACE2 protein remains controversial mainly because of discrepancies between antibodies. Importantly, we now know that there are 2 isoforms of ACE2, one of which is unable to bind to SARS-CoV-2. Future studies aimed at analyzing ACE2 mRNA and protein should attempt to determine which of the isoforms is detected considering the great impact this information will have on our understanding of SARS-CoV-2 infection and tropism. The distribution in respiratory tissues is heterogenous and ACE2 is mainly detected in ciliated, goblet, and club cells of the nose and bronchi and in the AT2 cells in the alveoli (Figure 1). The detection of ACE2 mainly in nasal cells could explain a higher shedding of virus in the upper respiratory tract at the beginning of the disease, which has been observed from repeated biological samples (Wang et al., 2020b; Zou et al., 2020). Conversely, the presence of ACE2 in AT2 might facilitate entry of SARS-CoV-2 as observed in infected macaques (Rockx et al., 2020) causing damages of alveoli that could contribute to COVID-19 severity (Olajuyin et al., 2019). Future studies should aim to determine if and how regulation of ACE2 expression during SARS-CoV-2 infection impacts the dynamics of infection and the antiviral response.

### ACE2 expression in comorbidities associated with COVID-19 severity: protective or harmful role?

A large body of evidence supports a protective role for ACE2 in several disease models. The role of ACE2 was initially described in the context of the renin-angiotensin system (RAS), with the discovery of the ACE2/Angiotensin (Ang 1–7)/Mas axis that counteracts the axis of the ACE/AngII/AT1 receptor. ACE2 also plays as a role in the regulation of the kallikrein-kinin system (KKS) that catalyzes the formation of the Bradykinin 2 (B2) receptor ligands, bradykinin and Lys-bradykinin, which are further hydrolyzed into the B1 receptor ligands des-Arg9-bradykinin and Lys-des-Arg9-bradykinin. ACE2 inactivates B1 receptor ligands by cleavage. Increased ACE2 levels, associated with a decrease in AngII due to conversion into Ang 1–7, is typically considered an indicator of organ protection because of its role in reducing pulmonary vasoconstriction, remodeling, atherosclerosis, blood pressure, myocardial hypertrophy, fibrosis and ventricular remodeling. Additionally, through inhibition of B1 receptor ligands, ACE2 is protective against pulmonary angioedema. On the contrary, a decrease in ACE2 levels is associated with an increase in pulmonary vascular permeability, pulmonary edema, ARDS, atherosclerosis, hypertension, cardiac hypertrophy, ventricular remodeling, and heart failure. Importantly, the ACE2/Ang 1–7/Mas axis and ACE2-dependent inhibition of B1R activation also exerts inhibitory effects on inflammation. These functions of ACE2 were fully reviewed by others (Muñoz-Durango et al., 2016; Simões e Silva et al., 2013; Mirabito Colafella et al., 2019; van de Veerdonk et al., 2020; Chung et al., 2020).

From the early days of the pandemic in China and Italy, epidemiological data rapidly indicated, in addition to the age of over 70 years, an association of COVID-19 disease severity, ICU admission and poor prognosis with multiple comorbidities, including hypertension and cardiovascular disease (CVD), COPD and type 2 diabetes (T2D) (Grasselli et al., 2020; Lippi and Henry, 2020; Guan et al., 2020; Huang et al., 2020; Jain and Yuan, 2020). Additionally, smoking is associated with COVID-19 disease progression (Gülsen et al., 2020). Reports arising notably from Europe and North America, have subsequently highlighted that a high BMI, and particularly obesity, is an important risk factor for a severe course of COVID-19 in patients younger than 60 years old (Kass et al., 2020; Soeroto et al., 2020). Although the factors responsible for these associations remain to be elucidated, questions concerning a potential role of ACE2 expression have emerged.

Expression of ACE2 in the pulmonary epithelium is well documented in COPD condition and relative to the smoking status. Elevated levels of ACE2 mRNA is observed in airway brushings from COPD patients compared to non-COPD and in current smokers vs never smokers (cohorts of n = 20–220) (Leung et al., 2020; Muus et al., 2020; Smith et al., 2020; Cai, 2020). Datasets from lung biopsies (n = 33–77 samples) also showed increased ACE2 mRNA levels in smokers in both Asian and Caucasian populations (Cai, 2020). These observations are confirmed by meta-analysis of pulmonary RNA-seq datasets from the GEO repository (Pinto et al., 2020). Although limited in the scope (n = 8 healthy, 9 smokers and 10 COPD donors), IHC detection of ACE2 (ab15348) in resected lung tissues supports higher protein expression in COPD, and to a lesser extent in smokers, compared to healthy individuals (Leung et al., 2020). Whether increased ACE2 expression in the lungs of COPD patients or active smokers (Figure 1) has a causal effect on the outcome of COVID-19 remains elusive. One could argue that increased ACE2 levels would allow more virus entry, as it happens in cell models transfected with hACE2 (Ou et al., 2020; Blanco-Melo et al., 2020), hence increased subsequent inflammatory response. This model is supported by the observation that patients (n = 76) with severe COVID-19 tend to have a high viral load and a long virus-shedding period (Liu et al., 2020a; Yu et al., 2020; Liu et al., 2020b). Alternatively, one can argue that through its role in the conversion of AngII, increased ACE2 in the lungs could be beneficial by protecting the lung from AngII-dependent lung injury and edema (Deng et al., 2012). Considering that lung injury and edema are clinical features of the most severe phase of COVID-19, a scenario where infection leads to increased levels of ACE2 is very unlikely. Rather, this hypothesis should be mitigated by the fact that SARS-CoV-2 triggers ACE2 downregulation and thereby the final outcome would be increased local AngII causing acute lung injury and edema. Potential implications of the RAS system and ACE2 in the lung have recently been fully reviewed (Chung et al., 2020; Samavati and Uhal, 2020). This latter hypothesis is consistent with a recently proposed model which argues that during infection with SARS-CoV-2, ACE2 would be less available to degrade the des-Arg9-bradykinin and Lys-des-Arg9-bradykinin peptides, thereby leading to overactivation of B1R and subsequently lung angioedema (van de Veerdonk et al., 2020). Future studies should interrogate the relationship between ACE2 levels in the lung and viral load and inflammation in smokers vs non-smokers and in COPD patients vs healthy individuals.

For comorbidities other than COPD, the correlation between the changes in ACE2 levels and the severity and prognosis of COVID-19 is less clear. Meta-analysis of 700 lung transcriptomes clearly highlighted the lack of data on the impact of hypertension and CVD on ACE2 expression in the lung (Pinto et al., 2020; Hamming et al., 2004). Given that cardiovascular complications are now well known to be a major threat to COVID-19 patients survival (Jain and Yuan, 2020), the question of the involvement of ACE2 located outside of the lung, particularly in the cardiovascular system, was raised. Expression of ACE2 at basal levels has been extensively reviewed elsewhere and therefore we focus here only on the description of ACE2 status in hypertension and CVD (Chung et al., 2020; Bourgonje et al., 2020). Increased ACE2 mRNA levels were observed in the ventricular myocardium from patients with idiopathic dilated cardiomyopathy (n = 11) or ischemic cardiomyopathy (n = 12) compared to healthy individuals (n = 9), and elevated ACE2 protein levels (IHC, anti-ACE2489) were found in explants from human ischemic hearts (Goulter et al., 2004; Burrell et al., 2005; Figure 1). Rats that suffer myocardial infarction also exhibited increased flACE2 mRNA and ACE2 protein levels and activity measured by emitted fluorescence with ACE2-specific quenched fluorescent substrate (Burrell et al., 2005). In patients presenting hypertensive nephrosclerosis (HTN, n = 41), a disease associated with chronic high blood pressure that induce kidney damage, ACE2 mRNA levels are decreased in the tubulointerstitium, but not in the glomerular zone, compared to healthy donors (n = 10) (Wang et al., 2011; Figure 1). Similarly, in spontaneously hypertensive rats (SHR), levels of ACE2 mRNA and protein were downregulated in kidneys (Tikellis et al., 2006). SHR also exhibit decreased levels of ACE2 protein (IB, sc-20998) in the rostral ventrolateral medulla (RVLM) containing cardiovascular regulatory neurons, a phenotype associated with the hypertensive state that is corrected by ACE2 injection (Yamazato et al., 2007). Salt-sensitive Sabra hypertensive (SBH) rats also showed decreased ACE2 mRNA and protein levels (IB, homemade antibody) in kidneys (Crackower et al., 2002). While there is a lack of data of ACE2 expression in hypertensive patients, animal models suggest a downregulation of mRNA and protein ACE2 in the kidney and RVLM. More investigations are needed to determine how ACE2 expression in specific organs in patients with hypertension and CVD could impact COVID-19 prognosis. The tropism of SARS-CoV-2 for cells in the cardiovascular system is a topic that attracts a lot of interest and is mostly supported by in-vitro infection of cardiomyocytes derived from induced pluripotent stem cells (Topol, 2020). Although a first report claimed post-mortem detection of viral inclusion in endothelial cells from three patients using electron microscopy (EM) (Varga et al., 2020), the interpretation of the images was subsequently challenged (Goldsmith et al., 2020). A thorough confirmation of this observation is necessary, in particular by using Immuno-EM with well validated antibodies. Importantly, demonstration of ACE2-dependent infection and replication of SARS-CoV-2 in endothelial cells are yet to be done. If this is confirmed, it will be important to determine whether the presence of viral structures in endothelial cells correlates with the observed increase in ACE2 (IHC, ab108252) observed post-mortem in pulmonary endothelial cells from COVID-19 patients (Ackermann et al., 2020). Response to this question will inform on the role of ACE2 in the endothelium. Finally, whether endothelial cells engagement is altered in patients with hypertension and CVD will need to be defined to determine if this could be a causal factor for the severity of COVID-19.

Increased levels of ACE2 in the lungs, as well as reduced levels in the vascular system, in T2D or obese patients and the possible mechanisms associated with the severity of COVID-19 have recently been reviewed (Kruglikov et al., 2020). ACE2 expression in the pancreas has also been under scrutiny, but results differ significantly between studies. While initial analysis of expression datasets suggested poor ACE2 mRNA and no detectable protein expression in the pancreas (Xu et al., 2020), a subsequent re-analysis of several datasets revealed expression in ductal cells (Muus et al., 2020). Similarly, analysis of two scRNA-seq datasets (n = 55 and n = 19) showed ACE2 expression in ductal and acinar cells on the exocrine gland, and to a lesser extent in beta cells of the pancreatic islet (Liu et al., 2020c). Moreover, ACE2 protein was detected (n = 20, IHC, HPA000288 and MAB933) in interlobular pancreatic ducts and endothelial cells (Hikmet et al., 2020). While in a mouse model of diabetes, ACE2 (IB, ab15347) was found upregulated in the liver and pancreas and pancreatic ACE2 activity was also increased (Roca-Ho et al., 2017), scRNA-seq analysis revealed decreased ACE2 mRNA levels in ductal cells of T2D patients compared to controls (Chen et al., 2020; Figure 1). If and how pancreatic ACE2 expression impacts the poor prognosis of COVID-19 remains to be elucidated. As of today, there is no report of SARS-CoV-2 infection in pancreatic cells. Considering that SARS-CoV N protein and RNA were detected in pancreatic acinar cells from 3 out of 4 SARS-CoV patients (Ding et al., 2004), the detection of SARS-CoV-2 in the pancreas should be considered in future investigations. Alternatively, the fact that diabetic patients are at high-risk of severe COVID-19 disease could be related to their overall elevated metabolic inflammation which may predispose to the cytokine storm syndrome associated with multi-organ failure (Bornstein et al., 2020). Excess of adipose tissue in T2D patients is associated with increased proinflammatory macrophages and Th1 and Th17 CD4+ T cells and is correlated to a chronic low-grade inflammatory state (Kulcsar et al., 2019). In addition, white adipose tissue produces proinflammatory cytokines, such as TNF, IL-1, IL-6 and IL-10 (Tsalamandris et al., 2019). Upon infection by SARS-CoV-2, the inflammatory state in T2D patients could be further exacerbated possibly leading to the cytokine storm and multi-organ failure (Bornstein et al., 2020).

Considering that the severity of COVID-19 is highly associated with an age over 70 years (except for obesity) assessing the role of age on ACE2 expression in patients with comorbidities in extensive retrospective epidemiological studies would provide a broader picture of risk factors and accurately inform on the potential consequences of modulating ACE2. Importantly, data collected from COVID-19 patients at the beginning of the pandemics were mostly cross-sectional and limited to the description of known underlying chronic diseases without stratification according to therapeutic treatment. Therefore, the question arose whether the observed association of comorbidities with COVID-19 severities might result from the treatments rather than the condition itself. Substantial data supports that RAS and ACE inhibitors and angiotensin-receptor blockers (ARBs), which are frequently used in patients with hypertension and CVD and to a lesser extent diabetes, induce increased expression of ACE2 as recently reviewed (Chung et al., 2020). Limited data supports that anti-diabetic treatments, Pioglitazone or Liraglutide, also increase ACE2 mRNA and protein (IB, ab108252) in liver and adipose tissue and in the lung and the heart, respectively (Pal and Bhadada, 2020; Zhang et al., 2014). The impact of corticosteroids or bronchodilators in COPD has to the best of our knowledge not yet been assessed. Overall, the impact of treatments on ACE2 remains to be fully explored in humans.

While evidence supports ACE2 increase in at least some comorbidities or in response to their associated treatments, it is not possible to conclude on the causality between ACE2 expression levels and the severity of COVID-19 (Pal and Bhadada, 2020; Vaduganathan et al., 2020). Currently available retrospective observational studies did not conclude on an association between the use of RAS inhibitors and increased risk of SARS-CoV-2 infection (Chung et al., 2020). A systematic meta-analysis of data from 15 studies, encompassing 2,065,805 COVID-19 patients with hypertension revealed that the probability of COVID-19 patients treated with RAS inhibitors to die was 35% less than those who were not under treatment (Ssentongo et al., 2020). Another meta-analysis of 9 studies including 3936 COVID-19 patients with hypertension showed that while there was no association between treatment with RAS inhibitor and the severity of the disease, patients under RAS treatment were less likely to die (OR 0.57, 95% CI 0.38–0.84, P0.004, I20) (Guo et al., 2020). Altogether, this suggests that even if RAS inhibitors induce ACE2 expression, they do not increase the risk for fatal COVID-19.

A possible alternative explanation that was recently discussed for comorbidities is that disfunction or absence of ACE2 may result in increased levels of AngII and hyperactivation of B1R which leads to inflammation, fibrosis, oxidative stress, vasoconstriction and angioedema (Chung et al., 2020). This could in fact be the case during infection with SARS-CoV-2 if virus-bound ACE2 is no longer available. In addition, while infection progresses, virus entry leads to the intake of ACE2, thereby resulting in a decreased amount of ACE2 at the membrane. Increased levels of AngII were found in the plasma of COVID-19 patients with pneumonia compared to healthy individuals and the levels of AngII positively correlated with SARS-CoV-2 viral load and lung injury (Liu et al., 2020b). It is possible that in patients presenting comorbidities associated with a dysregulation of the RAS system, AngII modulation by SARS-CoV-2 reaches a critical level that ultimately induces an inflammatory state causing fatal issues.

### Potential co-receptors/attachment factors for ACE2-dependent SARS-CoV-2 entry

The cell surface neuropilin NRP1 (also known as Vascular endothelial cell growth factor 165 receptor, VEGF165R Soker et al., 1997) and NRP2 (also known as VEGF165R2 Ambra et al., 2006) are dimeric receptors known to contribute to neurogenesis and angiogenesis (Yasuhara et al., 2004). They bind ligands containing a C-terminal polybasic motif that follows the C-end-Rule (CendR) (Teesalu et al., 2009). SARS-CoV-2 S contains a furin cleavage site which has the potential to generate a solvent exposed C-terminus containing the CendR R/KXXR/K sequence which is, according to molecular modeling predictions, capable of binding to the coagulation b1 domain of NRP (Teesalu et al., 2009; Cantuti-Castelvetri et al., 2020). This observation led to the hypothesis that neuropilins could serve as co-receptor for SARS-CoV-2 attachment to the cell surface (Figure 1) and contributes to the tropism as previously documented for viruses such as Human T-cell lymphotropic virus type 1 (HTLV-1) and Epstein–Barr virus (EBV) (Lambert et al., 2009; Wang et al., 2015). Several experimental observations support the role of NRP1 as SARS-CoV-2 co-receptor. In a GFP-nanotrap experiment in HEK293T cells, SARS-CoV-2 S1 interacts with GFP-NRP1 in a CendR motif-dependent manner (Daly et al., 2020). While deletion of NRP1 strongly reduced SARS-CoV-2 infection of Hela cells expressing ACE2, NRP1 alone was not sufficient as cells that do not express ACE2 are not infected by SARS-CoV-2 (*131*). In addition, incubation of VSV-SARS-S2 or patient-isolated SARS-CoV-2 with monoclonal anti-NRP1 decreased, although to a lesser extent than monoclonal anti-ACE2, the efficiency of infection of HEK293 cells expressing ACE2 and of Calu3 cells (Cantuti-Castelvetri et al., 2020; Daly et al., 2020). NRP1 and 2 are expressed in the respiratory epithelium with similar levels than ACE2 in AT2 cells. Cellular staining of NRP1 (IF, ab81321, n = 6) of post-mortem olfactory tissue sections showed high detection of NRP1 in infected epithelial cells of COVID-19 patients compared to control individuals (n = 7) (Cantuti-Castelvetri et al., 2020). Altogether, although still limited, current evidence suggests that NRP1 potentiates attachment of SARS-CoV-2 to facilitate ACE2-mediated entry. Further studies are required to determine if the use of neuropilins as co-receptors may partly explain that low levels of ACE2 in the respiratory epithelium are sufficient to allow efficient entry of SARS-CoV-2.

Heparan sulfate (HS), a negatively charged polysaccharide often found attached to proteins (proteoglycans) on the cell surface and extracellular matrix (Simon Davis and Parish, 2013), has been proposed to act as co-receptor of SARS-CoV-2. Differences in the structure and composition of HS between tissues is thought to define the tropism of some viruses (Cagno et al., 2019). Among coronaviruses, human coronavirus NL63 (HCoV-NL63) uses HS as a cell attachment point before binding to the ACE2 entry receptor (Milewska et al., 2014). In the context of SARS-CoV-2, molecular modeling highlighted a potential HS interaction site in the RBD domain of the S protein adjacent to the ACE2 binding hot spots (Clausen et al., 2020). Binding experiments confirmed that purified pseudotyped polinton-like viruses (pLV) expressing the S protein of SARS-CoV-2 bind immobilized HS (Tandon et al., 2020). While both the open and closed conformations of the RBD are capable of binding to HS, only the open one binds to ACE2. This points to a mechanism in which attachment of S to HS would trigger a conformational transition to stabilize the open conformation of SARS-CoV-2 S, thereby permitting the interaction with ACE2 (Clausen et al., 2020). The role of HS as attachment factor is further supported by the observation that modification of HS with bacterial glycosidases or degradation by HSase enzymes blocked SARS-CoV-2 infection of diverse cell lines and ALI-differentiated HBEC (Clausen et al., 2020; Martino et al., 2020). While SARS-CoV-2 infection sensitivity to HSase treatment was found to be independent of the cell type, HS isolated from human lungs bind more efficiently to the SARS-CoV-2 S RBD than HS from the kidneys (Clausen et al., 2020). Thus, whether HS contributes to SARS-CoV-2 tropism remains to be clarified.

Sialic acids have been described as cell attachment factors for many viruses including alpha- and betacoronaviruses (Böttcher-Friebertshäuser et al., 2014; Hulswit et al., 2016; Lim et al., 2016). Binding of MERS-CoV S to sialoglycoconjugates at the surface of erythrocytes and to α2,3-linked mono-sialotrisaccharides and α2,3-linked di-Sia and tri-Sia glycans, which are abundant in the trachea and the lungs, likely explains the tropism and transmission (Li et al., 2017; Wasik et al., 2017). Cryo-EM and crystallography showed that MERS-CoV S binds Neu5Ac sialic acids in a groove near to the binding site to the DPP4 entry receptor supporting a two-step binding mechanism (Park et al., 2019). A similar mechanism is suggested for murine coronavirus strain JHM infection that is sensitive to neuraminidase treatment and is enhanced upon expression of the entry receptor mouse carcinoembryonic antigen-related cell adhesion molecule (mCEACAM) (Qing et al., 2020). In the context of human coronavirus OC43 (HCoV-OC43) and human coronavirus HKU1 (HCoV-HKU1), binding of the S proteins to 9-O-acetylated sialic acid is sufficient to mediate entry in the host cell without requirement of other receptor (Zhou et al., 2020; Hulswit et al., 2019; Huang et al., 2015; Tortorici et al., 2019; Petrosillo et al., 2020; Milanetti et al., 2020). These observations led to the question of the potential role of sialic acids during SARS-CoV-2 infection. As of today, this remains speculative and mostly based on in-silico predictions and modeling. Sequence alignment and molecular modeling predict an interaction of SARS-CoV-2 S (243–302 aa) with sialic acids similar to the one observed for MERS-CoV S (Park et al., 2019; Milanetti et al., 2020; Vandelli et al., 2020). Further molecular dynamic simulation of SARS-CoV-2 S interaction with the ganglioside GM1, a glycosphingolipid containing one or more sialic acid residues (Fantini et al., 2020; Sántha et al., 2020; Kuhn and Wiegandt, 1963), points to the formation of a trimolecular complex between a ganglioside binding domain (111–162 aa) and two GM1 molecules. Although these studies propose distinct regions of interaction of S with sialic acids, they point to a model in which SARS-CoV-2 could bind to ganglioside regions exposed at the cell membrane potentially favoring the subsequent interaction of the RBD with ACE2 (Figure 1). Further in vitro and in vivo studies are necessary to determine if SARS-CoV-2 binding to sialic acids is required before interaction with ACE2. If so, this could be a major determinant of SARS-CoV-2 tropism.

Altogether, evidence supporting the involvement of NRP1 or HS as co-receptor/attachment factors favoring SARS-CoV-2 entry into cells is still sparse. This is even more true for the potential role of sialic acids that remains speculative. Further studies, ideally in vivo in animal models or in patients will be necessary to delineate the specific role of each of these elements in the control of SARS-CoV-2 infection and whether they constitute potential therapeutic targets to effectively inhibit the spread of the virus in the respiratory system. Whether the specific distribution of one or more of these factors in specific cell types could define SARS-CoV-2 tropism is a topic of interest. It is possible that different co-receptor/attachment factors bind to SARS-CoV-2 S at the same time without creating steric hindrance, but this remains to be assessed.

### Alternative receptors with potential role in SARS-CoV-2 entry

Despite convincing evidence that ACE2 is a high affinity receptor for SARS-CoV-2, the limited levels of expression throughout the respiratory system allow speculation on the uniqueness of this entry route. Data derived mainly from the study of other coronaviruses provide sufficient evidence to discuss the putative role of CD147 and GRP78 as entry receptors for SARS-CoV-2 (Figure 1). The Ang II type 2receptor (AGTR2), and ACE have also been proposed alternative receptors by others (Cui et al., 2020; Prates et al., 2020), but evidence is fairly weak and therefore will not be reviewed here.

The transmembrane glycoprotein CD147, also known as basigin or EMMPRIN (Miyauchi et al., 1990; Biswas et al., 1995), is ubiquitously expressed and mRNA levels are higher than those of ACE2 in the lung (Su et al., 2004; Wu et al., 2009). CD147 protein is also highly detectable by IHC (anti-CD147 detects only one band at the expected molecular weight by IB) in the airway epithelium of lung tissue sections (Aguiar et al., 2020). Regarding co-morbidities associated with the severity of COVID-19, expression of CD147 is increased in the respiratory mucosa in smokers and patients with COPD (Aguiar et al., 2020; Jouneau et al., 2011). CD147 plays a role in SARS-CoV infection which is blocked by the CD147 AP-9 antagonist peptide and by a humanized antibody against CD147 developed in-house (Meplazumab) (Wang et al., 2020c). This same antibody has been tested in 17 COVID-19 patients, 4 of which with moderate, 6 with severe and 7 with critical symptoms (Bian et al., 2020). Treatment with anti-CD147 enhanced virus clearance, as measured by PCR, and decreased lymphocytopenia and inflammation index measured by the presence of inflammatory foci in the lungs compared to patients hospitalized in the same period (n = 11). Large placebo-controlled study is still required to fully conclude on the efficiency of CD147 blockage to improve COVID-19 outcome. The role of CD147 as entry receptor in SARS-CoV-2 infection remains to be demonstrated, but SPR analyses showed a direct interaction between the RBD of the S protein and CD147 in vitro with a Kd of 185 nM (Wang et al., 2020c). This contrasts with the proposed model in SARS-CoV infection which postulates that the interaction between CD147 and the N protein is indirect and requires cyclophilin A (CyPA) (Chen et al., 2005). The interaction between CD147 and CyPA, which is ubiquitously expressed, is known to control the induction of inflammatory cytokines, such as TNF and IL-6 (Dawar et al., 2017a; Dawar et al., 2017b; Dai et al., 2016). Therefore, inhibiting CD147 during SARS-CoV-2 infection might also be a means of reducing the cytokine storm observed in critically ill patients. In conclusion, preliminary observations notably through patient treatment argue in favor of additional mechanistic studies to determine whether CD147 is involved in virus entry and defines novel tropism.

The ER chaperone BiP, also known as 78 kDa glucose-regulated protein (GRP78)/Binding-immunoglobulin protein (BiP)/Heat shock protein family A member 5 (HSPA5), is widely known for its role in the degradation of misfolded proteins and in the unfolded protein response (Yang et al., 2015; Ting and Lee, 1988; Baumeister et al., 2005; Werner et al., 1998). GRP78 is detected in bronchial epithelial cells and mRNA levels in the lung are significantly higher than ACE2 (Uhlén et al., 2015; Aguiar et al., 2020; Su et al., 2004; Wu et al., 2009). While GRP78 mainly resides in the ER, plasma membrane localization is also observed upon response to cellular stress (Ibrahim et al., 2019). In particular, GRP78 was found to relocalize to cell surface and serve as an attachment point for MERS-CoV and bat coronavirus HKU9 (bCoV-HKU9) (Chu et al., 2018). Complexes containing GRP78 and MERS-CoV S protein and colocalization of GRP78 and DDP4, the main entry receptor of MERS-CoV, have been observed. However, silencing of GRP78 only slightly reduced MERS-CoV entry compared to DDP4 pointing to a model in which GRP78 acts as a point of attachment potentiating virus entry via DDP4 (Chu et al., 2018). While there is no direct demonstration yet, the speculation that GRP78 could act as a receptor for SARS-CoV-2 came in part from the observation that the S protein contains disulfide bonds that form cyclic structures that share similarities to that of Pep42, a cyclic 13-mer (CTVALPGGYVRVC) that interacts with GRP78 at the cell surface (Kim et al., 2006). Modelization confirmed that SARS-CoV-2 S could stably interact with the head of GRP78 (Ibrahim et al., 2020). However, this study relies on the structure of S that differs from previously published structure obtained by cryo-EM in the number of disulfide bonds (Wrapp et al., 2020). Further mechanistic studies in vitro and in cells are needed to draw conclusions about the role of GRP78 in the entry of SARS-CoV-2.

### Concluding remarks

Clearly, a detailed characterization of receptor(s) and protease(s) involved in SARS-CoV-2 entry is of major importance to understand the tropism of SARS-CoV-2 and therefore the COVID-19 disease. Indeed, while in the early phase of the pandemic, SARS-CoV-2 was believed to behave like other respiratory viruses leading to ARDS, it is now very clear that it is a very unusual pathogen that provokes manifestations outside the respiratory apparatus leading to fatal outcomes in vulnerable people. Although research on receptors and proteases has highly benefited from accumulated knowledge on other human coronaviruses, notably the closely related SARS-CoV, much remains to be learned in the context of SARS-CoV-2 infection. The numerous data discussed in this review support a main role of ACE2 as for SARS-CoV. Although this is a plausible model, it is disputed by the observation that ACE2 is at most poorly expressed in the respiratory epithelium. Whether co-receptors, such as NRP1, HS or sialic acids, exposed at the surface of target cells could be part of the answer to define a two-step attachment mechanism should be further addressed. Research on alternative routes of entry is also worth pursuing. CD147 has emerged as a potential alternative entry receptor, but the fact that it is ubiquitously expressed raises questions about associated mechanisms that would be needed to explain the restricted tropism of SARS-CoV-2. With COVID-19 emerging as a disease with vascular consequences rather than just a respiratory syndrome, the identity of entry receptors is an even more important topic. Intriguing preliminary data suggesting infection of endothelial cells is consistent with the role of ACE2. However, more solid data, notably relying on post-mortem analyses or from relevant animal models, are urgently needed to validate this observation and determine whether it is anecdotic or more widespread among COVID-19 patients. Such data will have an immense impact on our understanding of COVID-19 pathogenesis and would undoubtedly guide future development of therapies. Finally, with the information currently available, it is very difficult to conclude on the role of the modulation of the entry receptors in patients with comorbidities associated with severe symptoms and fatal outcome of COVID-19. An important question awaiting answers is whether the involvement of ACE2 in SARS-CoV-2 infection has an impact on the RAS and KKS systems and whether this could be related to the inflammatory state of severe COVID-19.
